# TFEB Supports Pancreatic Cancer Growth through the Transcriptional Regulation of Glutaminase

**DOI:** 10.3390/cancers13030483

**Published:** 2021-01-27

**Authors:** Ji Hye Kim, Jinyoung Lee, Young-Ra Cho, So-Yeon Lee, Gi-Jun Sung, Dong-Myung Shin, Kyung-Chul Choi, Jaekyoung Son

**Affiliations:** Department of Biomedical Sciences, Asan Medical Center, AMIST, University of Ulsan College of Medicine, Seoul 05505, Korea; tym1503@ulsan.ac.kr (J.H.K.); wldndqke@ulsan.ac.kr (J.L.); youngla11@mail.ulsan.ac.kr (Y.-R.C.); meln922@naver.com (S.-Y.L.); kp9617@mail.ulsan.ac.kr (G.-J.S.); d0shin03@amc.seoul.kr (D.-M.S.); choikc75@amc.seoul.kr (K.-C.C.)

**Keywords:** TFEB, GLS, glutamine, PDAC

## Abstract

**Simple Summary:**

Pancreatic cancer is a highly lethal tumor with poor prognosis. In general, pancreatic cancer is not detected in its early stages since there are no signs or symptoms. A surgical resection gives the best chance for a cure, but these lesions are detected at the terminal or metastatic stages in most patients and surgery is therefore no longer feasible. New therapeutic options are thus necessary for the pancreatic cancer treatment. Alterations to metabolic pathways have recently attracted great interest as possible cancer treatments and many studies have reported that targeting glutaminase is an ideal approach in many cancers. Here, we provide reliable evidence that pancreatic cancer requires TFEB for maintaining glutaminase-mediated glutamine metabolism, and that this is an attractive new target for pancreatic cancer therapy.

**Abstract:**

Transcription factor EB (TFEB) is a master regulator of lysosomal function and autophagy. In addition, TFEB has various physiological roles such as nutrient sensing, cellular stress responses, and immune responses. However, the precise roles of TFEB in pancreatic cancer growth remain unclear. Here, we show that pancreatic cancer cells exhibit a significantly elevated TFEB expression compared with normal tissue samples and that the genetic inhibition of TFEB results in a significant inhibition in both glutamine and mitochondrial metabolism, which in turn suppresses the PDAC growth both in vitro and in vivo. High basal levels of autophagy are critical for pancreatic cancer growth. The TFEB knockdown had no significant effect on the autophagic flux under normal conditions but interestingly caused a profound reduction in glutaminase (GLS) transcription, leading to an inhibition of glutamine metabolism. We observed that the direct binding of TFEB to the GLS and TFEB gene promotors regulates the transcription of GLS. We also found that the glutamate supplementation leads to a significant recovery of the PDAC growth that had been reduced by a TFEB knockdown. Taken together, our current data demonstrate that TFEB supports the PDAC cell growth by regulating glutaminase-mediated glutamine metabolism.

## 1. Introduction

Pancreatic ductal adenocarcinoma (PDAC) is a type of exocrine pancreatic cancer and accounts for more than 90% of the diagnosed pancreatic cancers. It is a highly aggressive tumor with an expected 5-year survival rate of only ~8% [1]. This very poor prognosis is a consequence of a typically late stage diagnosis, which limits surgical intervention, as well as the resistance of PDAC tumors to conventional treatments by chemotherapy and radiation [2]. This is the principal reason why the 5-year survival rate of PDAC has not improved over the past few decades. Given their profound mortality rates and resistance to the available therapies, there is a strong impetus to identify new therapeutic targets for PDACs.

The MiTF/TEF family comprises four different transcription factors, namely microphthalmia-associated transcription factor (MITF), transcription factor E3 (TFE3), transcription factor EC (TFEC), and TFEB [3]. TFEB was the first member of the MiTF/TEF family identified as a master regulator of lysosomal biogenesis. It induces the transcriptional activation of genes implicated in lysosomal biogenesis, lysosomal acidification, lysosomal exocytosis, endocytosis, phagocytosis, and membrane repair [4]. Of note in particular, TFEB binds to the promoter of numerous autophagy genes and thereby activates the autophagosome and autolysosome formation [5]. TFE3 has also been shown to induce lysosomal biogenesis and autophagy. Cancer cells often have a restricted access to nutrients and oxygen as a result of their unlimited growth and insufficient blood flow. Autophagy is activated under nutrient-depletion or hypoxic conditions in order to optimize the response to metabolic stress. In regard to the activation of autophagy in cancer, overexpression of the MiTF/TEF family of transcription factors has been reported to play an essential role in tumorigenesis [6]. It has been reported also that PDAC tumors have an elevated expression of TFEB [7,8]. Among the characteristic features of PDACs is the increase in autophagy levels under basal conditions and the finding that the inhibition of autophagy suppresses the PDAC growth [9,10]. Hence, the MiTF/TEF family of transcription factors may be critical for maintaining high basal levels of autophagy, which is required for the PDAC tumorigenesis. Indeed, the MiTF/TEF transcription factors, most notably TFE3, coordinate the activation of lysosomal biogenesis and functions, which mediates the induction of autophagy [7]. However, another study has reported that the ability of TFE3 to activate the autophagy does not depend on TFEB and showed that the high levels of autophagy in PDAC is not decreased, even after a TFEB knockdown [8]. Thus, TFEB and TFE3 may respond to different types of physiological stresses and the role of TFEB in PDAC has remained largely unclear.

There has been growing evidence of a relationship between metabolism and cancer cell proliferation. Unlike normal cells, cancer cells exhibit significant increases in the uptake of glucose and the production of lactate, known as the Warburg effect [11]. Cancer cells undergo metabolic reprogramming in order to obtain sufficient amounts of the metabolites required for high rates of proliferation [12]. In addition to glucose, proliferating cancer cells also rely on glutamine. Cancer cells utilize glutamine as a mitochondrial substrate whereby it provides carbon to fuel the TCA cycle, and as a primary nitrogen donor in nucleotide and amino acid biosynthesis [13]. In addition, glutamine is an important amino acid in the maintenance of redox homeostasis by producing NADPH through glutaminolysis [14]. Hence, many cancers which rely on glutamine metabolism for their growth and survival have been reported to be dependent on glutamine [15]. For this reason, targeting glutamine metabolism may be a viable therapeutic approach for cancers that exhibit glutamine dependence. 

In our present study, we demonstrate that pancreatic cancers have high levels of TFEB, and that this is indispensable for their growth. TFEB transcriptionally regulates the GLS expression by directly binding the GLS gene promoter. The genetic suppression of TFEB inhibits the glutamine metabolic pathways that plays a critical role in the PDAC growth. These findings may have implications for future therapeutic approaches involving the inhibition of glutamine metabolism in PDAC tumors.

## 2. Materials and Methods

### 2.1. Cell Culture and Reagents

All of the human pancreatic cancer cell lines used in this study were sourced from the American Type Culture Collection and were tested regularly for mycoplasma contamination. All cells were incubated at 37 °C in humidified air with 5% CO_2_ and grown in Dulbecco’s modified Eagle’s Medium (DMEM; Thermo Scientific, Waltham, MA, USA) supplemented with 10% fetal bovine serum (FBS), 100 U/mL penicillin, and 100 μg/mL streptomycin (Thermo Scientific). 

### 2.2. Cell Growth Assay

For growth assays, cells were plated in 24-well plates at 2000 cells per well. The culture medium was not changed throughout the course of the experiment. At the indicated time intervals, the cells were fixed in 10% formalin and stained with 0.1% crystal violet. The dye was extracted with 10% acetic acid, and relative proliferation levels were determined according to the optical density at 595 nm.

### 2.3. Colony Formation Assay

For colony formation assays, the cells were plated in 6-well plates at 500 cells per well in a growth medium. The medium was not changed throughout the course of the experiment. After 7–10 days, the cells were fixed in 80% methanol and stained with a 0.2% crystal violet.

### 2.4. Quantitative Real-Time PCR

RNA was isolated with TRIzol (QIAGEN, Hilden, Germany) and cDNA was synthesized using 2 µg of RNA with MMLV HP reverse transcriptase (Epicentre, Madison, WI, USA). The quantitative real-time PCR was performed with a SYBR Green dye using an AriaMx Real-Time PCR system (Agilent Technologies, Santa Clara, CA, USA). The relative amounts of cDNA were calculated via the comparative Ct method using 18S ribosomal RNA sequences as a control. The GLS primer sequences were as follows: Forward, GCTGTGCTCCATTGAAGTGA; reverse, GCAAACTGCCCTGAGAAGTC.

### 2.5. Metabolomics

A targeted LC-MS/MS metabolomic analysis was performed as previously described [16]. Briefly, the cells were grown to ~60% confluence in a growth medium on 10 cm dishes in biological triplicates. After 24 h, the cells were harvested using 1.4 mL of cold methanol/H_2_O (80/20, *v*/*v*) after washing with a phosphate-buffered saline and water several times as well as lysis by vigorous vortexing; 100 μL of a 5 μM internal standard was then added. The metabolites extraction was then performed using a liquid–liquid extraction with chloroform. The aqueous phase was dried using a vacuum centrifuge, and the sample was dissolved in 50 μL of 50% methanol prior to the LC-MS/MS analysis. 

### 2.6. Transcript Analysis of Clinical mRNA Microarrays

The expression levels of TFEB in a normal and pancreatic cancer tissue were analyzed using the Oncomine platform (https://www.oncomine.org/), an online cancer microarray database. The threshold was determined according to the following values: *p*-value, 1 × 10^−4^; fold change, 2; and gene ranking in the top 10%. The differences in the RNA expression levels of TFEB and between normal and pancreatic cancer tissues were evaluated using the gene expression profiling interactive analysis (GEPIA) (http://gepia.cancer-pku.cn/). 

### 2.7. Measurement of the Oxygen Consumption Rate

The measurement of the oxygen consumption rate was performed as previously described [16]. Briefly, the cells were seeded in an XF24 cell culture microplate and cultured at 37 °C with 5% CO_2_. After 24 h, the culture medium was replaced with an unbuffered DMEM, and the cells were incubated at 37 °C without CO_2_ for 1 h. For measuring OCR, the cells were exposed sequentially to oligomycin (2 μM), FCCP (5 μM), and rotenone (2 μM).

### 2.8. Luciferase Assay

One day before transfection, the cells were plated at a density of 1~2 × 10^5^ cells per well in a 12-well plate in 1 ml of growth media to achieve 90~95% confluence at the time of transfection. The cells were transfected, using Lipofectamine 2000 (Thermo Scientific, Waltham, MA, USA) and serum free media, with 600 ng of the GLS promoter and control plasmids and 1 µg of β-gal DNA. Cells were incubated at 37 °C in a CO_2_ incubator for 5 h, after which the medium was replaced with a complete medium. The cells were then incubated at 37 °C in a CO_2_ incubator for 18–24 h prior to testing for the transgene expression. The cells were harvested and lysed using a RIPA lysis buffer containing a protein inhibitor cocktail. Luciferase activities were measured using a commercial Luciferase Assay System (Promega, Madison, WI, USA).

### 2.9. Chromatin Immunoprecipitation (ChIP) Assay

A Pierce Magnetic ChIP Kit (Thermo Scientific) was used to conduct the ChIP assays in accordance with the manufacturer’s protocol. Briefly, the cells were treated with temozolomide (100 μM) for 72 h followed by fixation in 1% paraformaldehyde and incubation with glycine. The cells were then lysed and subjected to the MNase digestion. The lysates were sonicated to obtain DNA fragments with shear lengths of 200–1000 bp using three sets of 20 s sonication pulses. The anti-normal Rabbit IgG included in the kit or anti-TFEB (Bethyl Laboratories, Montgomery, TX, USA) antibodies were used to precipitate the DNA-protein complexes. The immunoprecipitated DNA was then examined by PCR. Primers specific for GLS mRNA were used as follows: Sense, 5’-GGGTTAAATTTCAGTGGACTC-3’; antisense, 5’-CAGGACAATGGGAAAGTACTTAG-3’.

### 2.10. Xenograft Studies

Male severe combined immunodeficiency mice were purchased from Joong Ah Bio. All the experimental procedures were approved by the Institutional Animal Care and Use Committee of Asan Institute for Life Sciences (2020-02-138). For subcutaneous xenografts, 8988T cells were infected with lentiviral shRNAs targeting TFEB (n = 2) and GFP (control hairpin, n = 1) and subjected to a short period of puromycin selection (2 μg/mL). A total of 1 × 10^6^ cells, suspended in 100 μL of Hank’s Buffered Saline Solution, were injected subcutaneously into the lower flank of each mouse (four mice per group). The tumor length and width were measured twice weekly, and the volume was calculated according to the following formula: (Length × width^2^)/2.

### 2.11. Lentiviral-Mediated shRNA Targets

The following RNA interference (RNAi) consortium clone IDs for shRNAs were used in this study: TRCN0000013112 (shTFEB-1; TGATCCACTTCTGTCCACCAT) and TRCN0000013109 (shTFEB-2; CGATGTCCTTGGCTACATCAA).

### 2.12. Statistics

Data are presented as a mean value ± standard deviation. All the comparisons were statistically analyzed using an unpaired Student’s t-test.

## 3. Results

### 3.1. TFEB Is Essential for PDAC Growth

**Figure 1:**
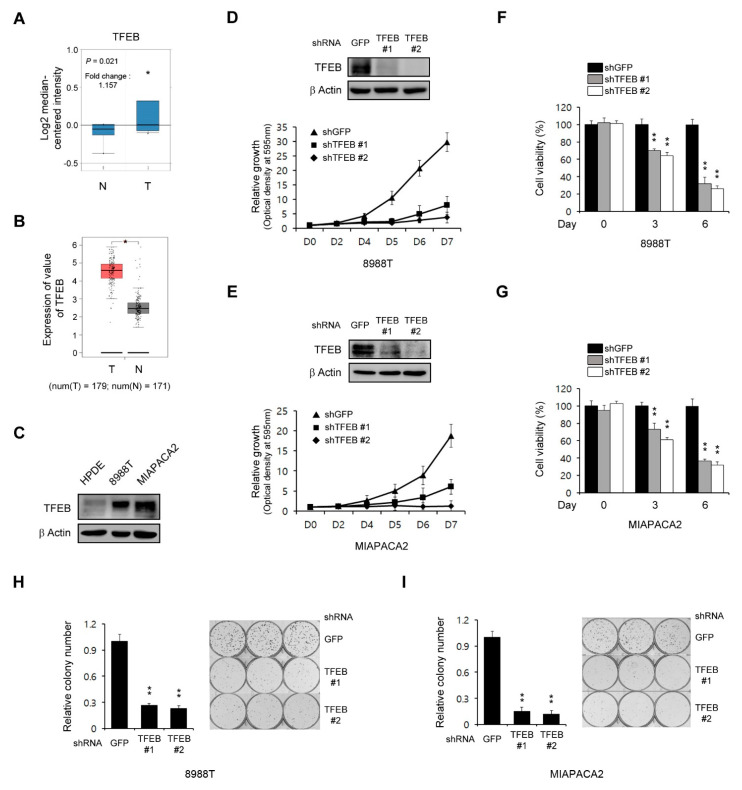
Transcription factor EB (TFEB) knockdown reduces the pancreatic ductal adenocarcinoma (PDAC) growth. (**A**) Box plots derived from the gene expression data in Oncomine comparing the expression of the TFEB gene in six normal tissue samples (left plot) and in 11 pancreatic cancer samples (right plot). (**B**) TFEB gene expression was detectable in 179 pancreatic cancer tissues (T) and 171 normal tissues (N) from the gene expression profiling interactive analysis (GEPIA) database. (**C**) Immunoblots of the TFEB expression in human pancreatic ductal (HPDE) and PDAC cells. (**D**,**E**) Cell growth assay for PDAC cells infected with shRNAs against GFP or TFEB. Western blot analysis confirmed the knockdown of TFEB expression. (**F**,**G**) Cell viability assay for PDAC cells infected with shRNAs against GFP or TFEB. (**H**,**I**) Clonogenic assay for PDAC cells infected with shRNAs against GFP or TFEB. Error bars represent the standard deviation (SD) of triplicate wells from a representative experiment; * *p* < 0.05; ** *p* < 0.01. (Please find western blot in Supplementary files)

As the role of TFEB in tumor growth is complex and dependent on the tumor type, we evaluated the functions of this transcription factor in the PDAC growth. To address this, we first examined the expression of TFEB in human pancreatic cancer using the Oncomine database (http://www.oncomine.org). The expression of TFEB was found to be higher in pancreatic cancer samples compared with normal tissue samples (Figure 1A). To further validate the high levels of TFEB in pancreatic cancer tissues compared with the normal tissues, we used the GEPIA database (http://gepia.cancer-pku.cn/) and found that the expression of TFEB was higher in 179 pancreatic cancer tissues compared with 171 normal tissues (Figure 1B). Consistent with these data, PDAC cells exhibited elevated levels of TFEB expression compared with non-transformed human pancreatic ductal (HPDE) cells (Figure 1C). 

We next explored the effect of TFEB in the PDAC growth through the downregulation of TFEB expression using RNAi. As shown in Figure 1D,E, this TFEB knockdown significantly inhibited the PDAC growth. To confirm this finding, we next explored cell viability upon the TFEB knockdown. Consistent with the growth curve, the TFEB knockdown led to a significant inhibition in the PDAC proliferation (Figure 1F,G). Additionally, the TFEB knockdown also inhibited the clonogenic growth of PDAC cells (Figure 1H,I). Therefore, TFEB was demonstrated to be essential for the PDAC growth. 

### 3.2. TFEB Is Not Required for the High Basal Levels of Autophagy in PDAC Cells

**Figure 2:**
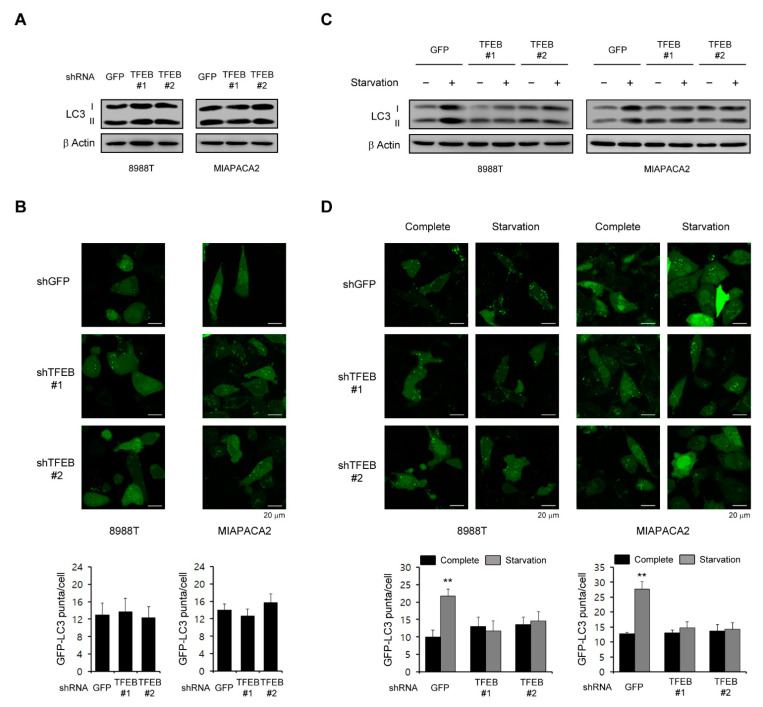
Effects of TFEB on the basal levels of autophagy. (**A**) PDAC cells infected with shRNAs against GFP or TFEB were immunoblotted with the indicated antibodies. (**B**) PDAC cells expressing the control shRNA (shGFP) or shRNAs targeting TFEB were infected with a lentivirus expressing GFP-LC3 and analyzed for LC3 dots. (**C**,**D**) PDAC cells infected with shRNAs against GFP or TFEB were plated in compete media, which was replaced the following day with glutamine-free media. (**C**) The cells were immunoblotted with the indicated antibodies 48 h after supplementation with glutamine free media. (**D**) The cells were infected with a lentivirus expressing GFP-LC3 and analyzed for LC3 dots 48 h after supplementation with glutamine free media. ** *p* < 0.01. (Please find western blot in Supplementary files)

The role of autophagy in tumorigenesis is complex and is likely to be dependent on the tumor type [17], but many studies have reported that autophagy plays an important role in the PDAC growth as a tumor promoter [9,18]. Therefore, we speculated that TFEB supports the PDAC growth by maintaining high basal levels of autophagy since it is a master transcription factor in the autophagic response. Interestingly however, a TFEB knockdown had no significant effect on the LC3-II levels in PDAC cells (Figure 2A). To further confirm the effect of TFEB on autophagy, we examined the recruitment of LC3 into autophagosomes using a GFP-LC3 reporter. Consistent with the lack of an effect of the TFEB knockdown on LC3-II levels, there was no significant change in the number of GFP-LC3 puncta upon the TFEB knockdown (Figure 2B). 

Given that TFEB induces autophagy in response to stress conditions such as nutrient limitation [7,19], we speculated that this transcription factor may be important for the activation of autophagy upon nutrient starvation. Indeed, as shown in Figure 2C, autophagy was activated following nutrient starvation in PDAC cells expressing control shRNA (shGFP), whereas there was no significant increase in LC3-II levels upon the TFEB knockdown. Additionally, the number of GFP-LC3 puncta was not increased upon the TFEB knockdown compared with the cells expressing a control shGFP (Figure 2D). Taken together, these data suggested that TFEB is necessary for autophagy activation under stress conditions but not under normal conditions. 

### 3.3. TFEB Knockdown Has a Potent Effect on Mitochondrial Metabolism

**Figure 3:**
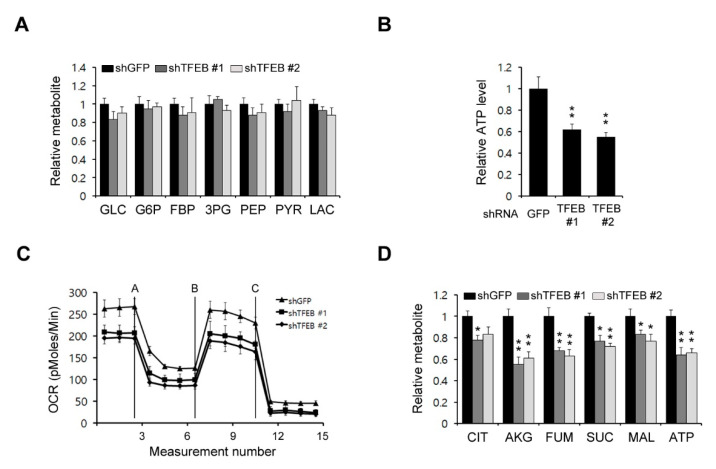
TFEB inhibits mitochondrial metabolism. (**A**) Glycolysis metabolite pools in 8988T cells infected with shRNAs against GFP or TFEB were analyzed via LC-MS/MS. (**B**) The 8988T cells infected with shRNAs against GFP or TFEB were analyzed for intracellular adenosine triphosphate (ATP). (**C**) Oxygen consumption rates in 8988T cells infected with shRNAs against GFP or TFEB were measured with an extracellular flux analyzer. (**D**) TCA (tricarboxylic acid cycle) metabolite pools in 8988T cells infected with shRNAs against GFP or TFEB were analyzed via LC-MS/MS. Error bars represent the SD of triplicate wells from a representative experiment; * *p* < 0.05; ** *p* < 0.01.

As cancer cells rewire their metabolic pathways to meet the energetic and biosynthetic demands of their high rates of proliferation, they rely heavily on alterations to these pathways for their growth. We assessed the possibility that a TFEB knockdown may inhibit some metabolic pathways, which in turn would reduce the PDAC growth. To examine the functional role of TFEB in PDAC metabolism, we first investigated the effect of TFEB on glucose metabolism via the targeted liquid chromatography-tandem mass spectrometry (LC-MS/MS) metabolomic analysis of PDAC cells. As shown in Figure 3A, a TFEB knockdown had no significant effect on glycolysis metabolites. We next investigated the effect of TFEB on mitochondrial metabolism. A TFEB knockdown resulted in a significant reduction in the adenosine triphosphate (ATP) levels (Figure 3B). The oxygen consumption rate also decreased markedly upon the TFEB knockdown (Figure 3C). To further confirm the importance of TFEB for mitochondrial metabolism, we next performed a metabolic analysis. As shown in Figure 3D, the TFEB knockdown resulted in significant decreases in the TCA intermediate levels. These results demonstrated that TFEB is critical for mitochondrial metabolic functions and energy levels.

### 3.4. TFEB Plays an Important Role in Glutamine Metabolism

**Figure 4:**
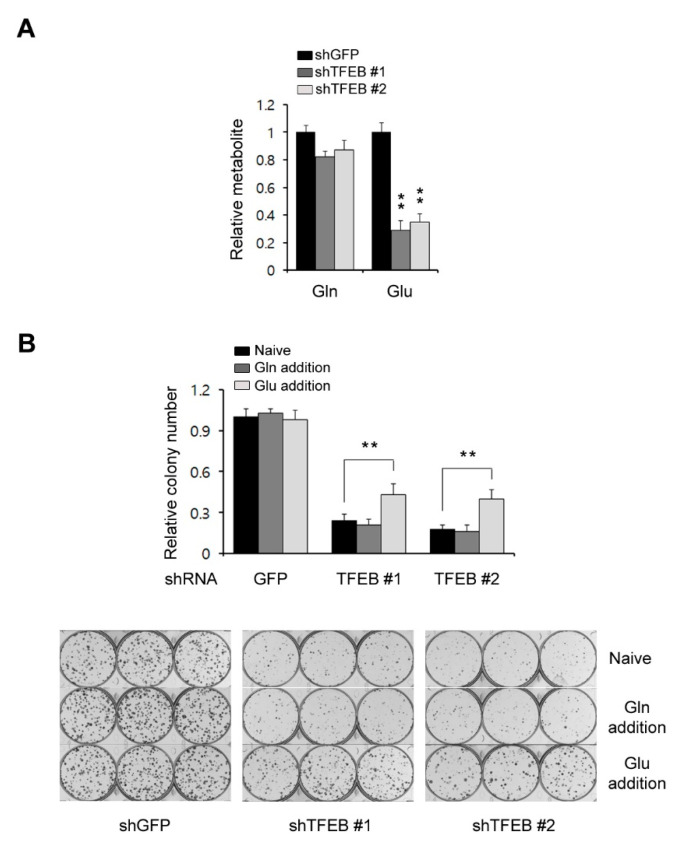
TFEB knockdown reduces the glutamate levels. (**A**) Glutamine levels in 8988T cells infected with shRNAs against GFP or TFEB were monitored using LC-MS/MS. (**B**) Relative clonogenic growth of 8988T cells infected with shRNAs against GFP or TFEB was analyzed by plating the cells in complete media supplemented with either glutamine (4 mM) or glutamate (4 mM). Error bars represent the SD of triplicate wells from a representative experiment; ** *p* < 0.01. Gln: Glutamine; Glu: Glutamate.

Glucose is a major source of energy and biosynthesis in proliferating tumor cells [20]. In addition to glucose, many tumor cells depend on glutamine to meet their biosynthetic needs [21]. In general, tumor cells utilize either glucose or glutamine to fuel the TCA cycle and the utilization of either as a key carbon source for this purpose depends on the cancer type [22]. It has been reported that glutamine is a major carbon source for the TCA cycle in KRas G12D-driven PDAC cells [23]. In addition, our current results have demonstrated that a TFEB knockdown significantly reduces the levels of TCA cycle intermediates, the oxygen consumption rates, and the ATP levels, but does not cause dramatic decreases in glycolysis metabolites. Therefore, we speculated that TFEB may influence glutamine metabolism and assessed both the glutamine and glutamate levels. A TFEB knockdown had no significant effect on the glutamine levels but the glutamate levels were markedly decreased (Figure 4A). To test whether the TFEB knockdown-mediated growth inhibition is the result of insufficient glutamate levels, we attempted to rescue the TFEB knockdown-mediated growth inhibition by supplementing the cells with either glutamine or glutamate. We observed that glutamate but not glutamine supplementation rescued the cells from the TFEB knockdown-mediated growth inhibition (Figure 4B). These data indicated that TFEB may be required to maintain the intracellular levels of glutamate in PDAC tumors. 

### 3.5. TFEB Regulates Glutaminase Expression

**Figure 5:**
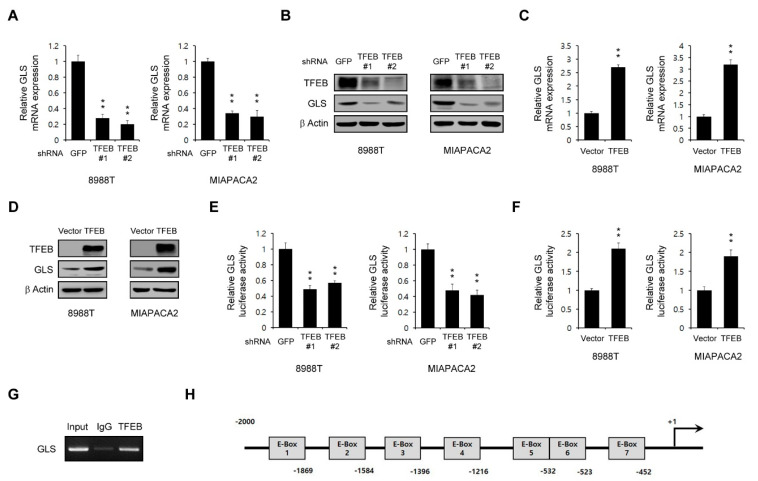
TFEB transcriptionally controls the glutaminase (GLS) expression. (**A**) GLS expression in PDAC cells infected with shRNAs against GFP or TFEB was determined by the quantitative RT-PCR analysis. (**B**) Effects of a TFEB knockdown on the GLS protein levels in PDAC cells infected with shRNAs against GFP or TFEB. (**C**) GLS was assayed by the quantitative RT-PCR analysis of PDAC cells expressing pCDH (vector) or pCDH-TFEB (TFEB). (**D**) PDAC cells expressing pCDH (vector) or pCDH-TFEB (TFEB) were immunoblotted with the indicated antibodies. (**E**) Luciferase expression verification of the reporter gene expression in PDAC cells infected with shRNAs against GFP or TFEB. (**F**) Luciferase expression verification of the reporter gene expression in PDAC cells expressing pCDH (vector) or pCDH-TFEB (TFEB). (**G**) The 8988T cells expressing TFEB were immunoprecipitated using a ChIP assay with anti-TFEB or anti-IgG antibodies. GLS gene expression was then analyzed by RT-PCR. (**H**) Schema for the cloned promoter region of human GLS. The putative TFEB-binding sites (E-boxes) are indicated. Error bars represent the SD of triplicate wells from a representative experiment; ** *p* < 0.01. (Please find western blot in Supplementary files).

Glutamate is mainly produced by glutaminase (GLS), which facilitates the conversion of glutamine to glutamate [24]. Pancreatic cancers display a dependence on glutamine metabolism for their growth [14]. Given that the intracellular levels of glutamate are significantly decreased upon the TFEB knockdown, we speculated that TFEB might control the GLS expression. Therefore, we tested whether a TFEB knockdown inhibits the GLS mRNA levels. As shown in Figure 5A, this TFEB knockdown resulted in a marked decrease in the GLS expression at the transcriptional level. Consistent with this result, the protein levels of GLS were also reduced upon the TFEB knockdown (Figure 5B). In addition, we observed that the overexpression of TFEB led to a significant increase in both its mRNA and protein levels (Figure 5C,D). 

For further confirmation of the transcriptional role of TFEB in the GLS expression, an approximately 2 kb fragment upstream of the GLS transcription start site was fused to the luciferase gene. We found that the luciferase activity was significantly reduced upon the TFEB knockdown (Figure 5E) and increased upon the TFEB overexpression (Figure 5F). We next performed a chromatin immunoprecipitation (ChIP) assay to determine the binding of TFEB to the GLS gene promoter. As shown in Figure 5G, the overexpression of TFEB significantly increased this binding. The promoter analysis of the 2 kb fragment located near the transcription start site of GLS revealed seven potential E-boxes within this region (Figure 5H). 

### 3.6. TFEB Knockdown Suppresses Tumor Growth

**Figure 6:**
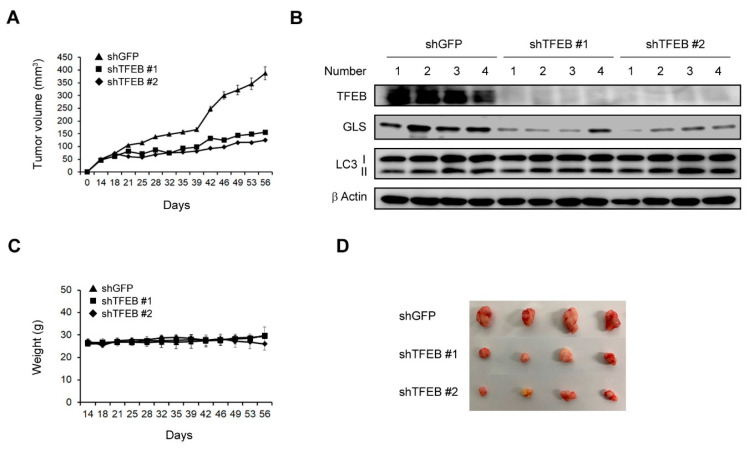
Effects of TFEB on the tumor growth in vivo. (**A**) Xenograft growth of 8988T cells infected with shRNAs against GFP or TFEB in mice. Error bars represent the SEM (n = 4). (**B**) Xenograft tumors were collected from the animals and tissue lysates were immunoblotted with the indicated antibodies. (**C**) Record of the mouse body weights. (**D**) Representative images of the xenograft tumors obtained from the mice. (Please find western blot in Supplementary files).

To examine the effects of TFEB on tumor growth, we subcutaneously injected 8988T cells infected with shRNAs against GFP or TFEB into mice to construct a xenograft mouse model. The TFEB knockdown led to a significant inhibition of the tumor growth (Figure 6A). Xenograft tumors generated from human PDAC cells infected with shRNA against TFEB were collected and tissue lysates were immunoblotted with the indicated antibodies. Consistent with our in vitro findings (Figure 2A and Figure 5B), the TFEB knockdown had no significant effect on the LC3-II levels and resulted in a significant reduction in the protein levels of GLS (Figure 6B). Moreover, this knockdown had no significant effect on the body weights of the mice, indicating that the downregulation of TFEB did not induce obvious toxicity (Figure 6C). We also found that the size of the xenograft tumors was dramatically reduced following the TFEB knockdown (Figure 6D). These findings indicated that TFEB is essential for tumorigenesis in vivo.

## 4. Discussion

Our present study findings demonstrate that TFEB transcriptionally regulates the glutaminase expression, which is essential for glutaminolysis, and that the suppression of this transcription factor leads to a significant reduction in glutamate levels, which in turn inhibits the PDAC growth in vitro and in vivo. 

Growing evidence has indicated the bifunctional roles of autophagy in the cellular homeostasis of cancer [25]. It is well known that autophagy is required by cancer cells to adapt to and survive different stress conditions such as nutrient limitation, protein misfolding, oxidative stress, or organelle damage. Cancer cells also require a constitutive basal level of autophagy to maintain cellular homeostasis for tumorigenesis [26] and nonconventional energy sources which are mobilized through nutrient scavenging pathways such as autophagy to support their high rates of proliferation [27,28]. Thus, many cancers are highly dependent on the constitutive activation of autophagy, which degrades and recycles cellular materials to sustain the metabolic function and energy homeostasis [29,30]. PDAC cells exhibit these high basal levels of autophagy as part of their growth requirements [31]. Hence, these cells require certain factors such as oncogenes to induce the constitutive activation of autophagy under normal conditions. 

The MiT/TEF transcription factors play an important role in the regulation of autophagosome biogenesis [32]. One member of this family, TFEB has been shown under stress conditions to control the expression of several genes involved in autophagosome initiation, elongation, and substrate capture, and also in autophagosome trafficking and fusion with lysosomes [5,33]. These studies have demonstrated the stress-adaptive roles of the MiT/TEF transcription factors. Recently, a number of studies have reported that the aberrant regulation of MiT/TEF transcription factors plays a critical role in tumorigenesis in a similar manner to oncogenes [6,7,34]. Consistent with our current results, PDAC has been shown previously to exhibit an elevated expression of MITF, TFE3, and TFEB [7,8]. However, one of these prior studies reported that MITF and TFE3 but not TFEB are major regulators of the transcriptional control of autophagy in PDAC cells [7]. Another study found that the autophagic flux is maintained following the TFEB knockdown [8] and it was reported also that oncogene Ras upregulates basal autophagy [35]. Consistent with these previous findings, our current data have indicated that TFEB is not required for high basal levels of autophagy in PDAC cells (Figure 2). Therefore, TFEB may have an alternative role if it is not an autophagy activator in PDAC cells. Indeed, our present data demonstrate that TFEB plays an essential role in glutaminolysis by transcriptionally regulating GLS. 

GLS has been shown to be regulated transcriptionally by either transcription factors or miRNAs. The c-Myc protein downregulates miR-23a and miR-23b, which enhances the expression of GLS [36]. The mTORC1 signaling positively regulates GLS by enhancing c-Myc translation [37]. Other miRNAs including miR-153 and miR-1-3p also regulate the GLS expression [38]. Our present data have revealed that the oncogenic transcription factor TFEB transcriptionally regulates GLS by directly binding to its gene promoter (Figure 5G). TFEB recognizes and binds a common DNA hexanucleotide sequence (CACGTG), known as an E-box that is commonly found in the promoters of target genes [3]. We found that there are seven E-boxes in the promoter regions of GLS, which can potentially be regulated by TFEB. Therefore, our results indicate that TFEB may regulate glutamine metabolism by transcriptionally controlling GLS. 

Many cancers including PDACs rewire their metabolic pathways in order to rely more strongly on glutamine for their survival and proliferation [38]. Therefore, many cancer cell types depend heavily on the elevated glutaminolysis, which converts glutamine into TCA cycle metabolites in a metabolic pathway that involves the initial deamination of glutamine by GLS, the most important enzyme in glutaminolysis [39]. Thus, GLS has become a potentially ideal therapeutic target in cancer. However, although several specific GLS inhibitors have been developed, only one has entered clinical trials to date [40]. Therefore, much research is still focused on finding targets to control the GLS activity. Here, we provide reliable evidence for the oncogenic role of TFEB in PDAC. The genetic inhibition of TFEB results in the significant suppression of the PDAC cell proliferation in vitro and in vivo. TFEB directly interacts with the promoter of GLS and regulates glutaminolysis. Our study is the first to report a GLS regulatory mechanism that involves TFEB. This transcription factor may be an attractive target to control the GLS activity as a therapeutic strategy in cancers with a high dependency on glutamine.

## 5. Conclusions

This study provides reliable evidence that TFEB is required for the PDAC growth by regulating TFEB-mediated glutaminolysis. Pancreatic cancer cells show a significantly elevated TFEB expression compared with normal tissue samples. Notably, TFEB transcriptionally controls the GLS expression by directly binding to the GLS gene promotor and regulates glutaminolysis, which is critical for the PDAC growth. The genetic suppression of TFEB leads to a significant inhibition in glutamine metabolic pathways, which in turn suppresses the PDAC growth both in vitro and in vivo.

## Data Availability

The data presented in this study are available from the corresponding author on a reasonable request.

## Supplementary Materials

The following are available online at https://www.mdpi.com/2072-6694/13/3/483/s1, Supplementary files: The western blot of Figure 1, Figure 2, Figure 5 and Figure 6.
