# Supplementary material for: TFEB Supports Pancreatic Cancer Growth through the Transcriptional Regulation of Glutaminase
Source: Cancers (Basel). 2021 Jan 27;13(3):483. doi: 10.3390/cancers13030483 (PMC7865852; doi:10.3390/cancers13030483)
Supplement: Supplementary file 1 [file cancers-13-00483-s001.pdf]

Fig 1

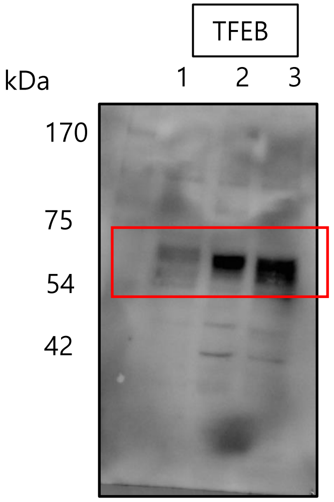

100: 276.3: 244

Fig 1C TFEB

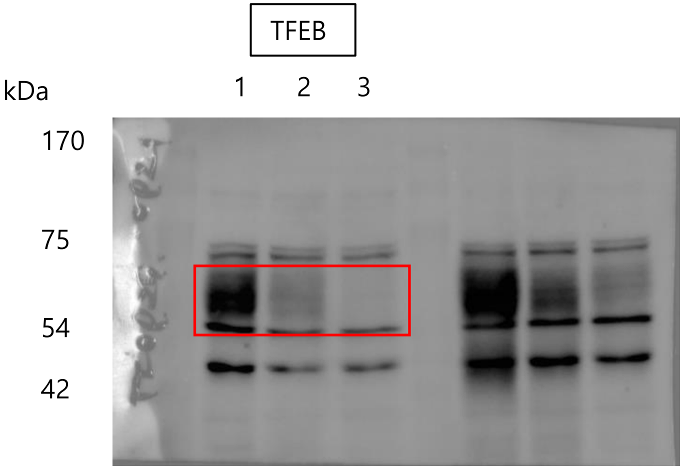

100: 20.6: 4.6

Fig 1D TFEB (8988T)

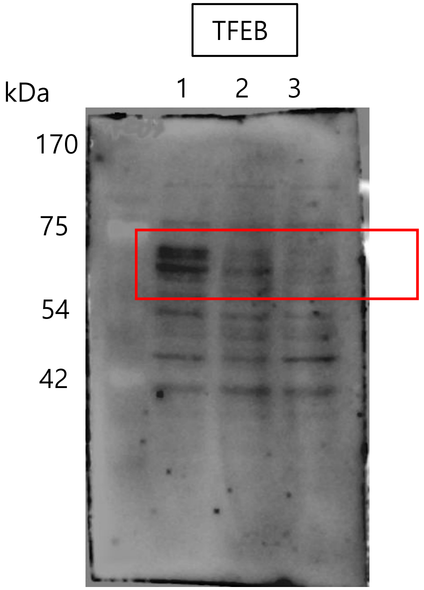

100: 50.4: 44.4

Fig 1E TFEB (MIAPACA2)

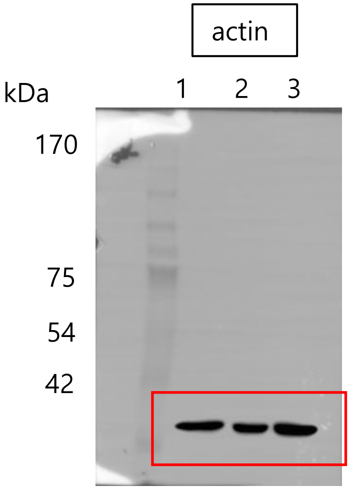

100: 121.2: 116

Fig 1C Actin

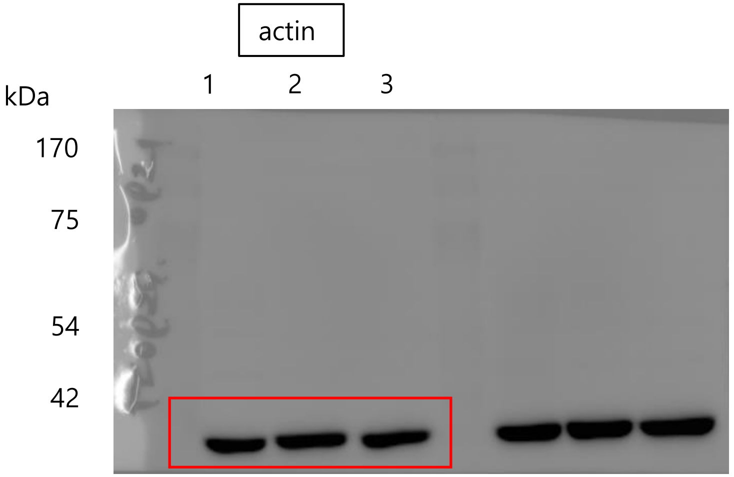

100: 72.3: 108.7

Fig 1D Actin (8988T)

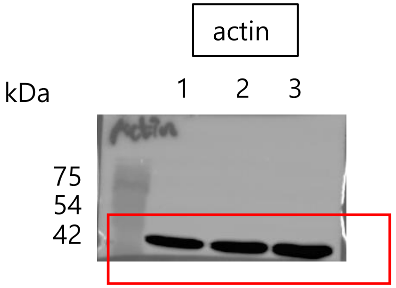

100: 119.2: 160.8

Fig 1E Actin (MIAPACA2)

Fig 2

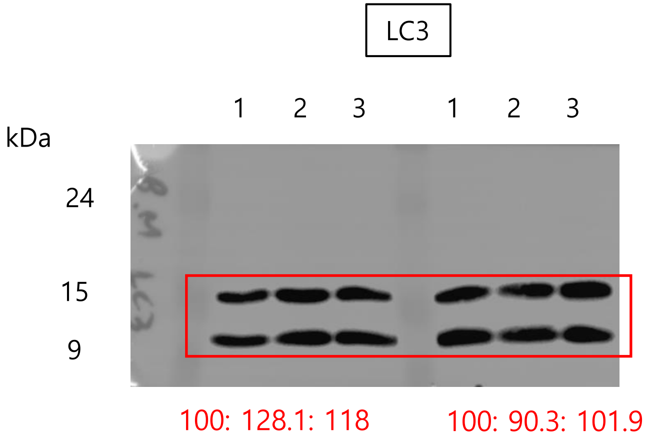

Fig 2A LC3 (8988T, MIAPACA2)

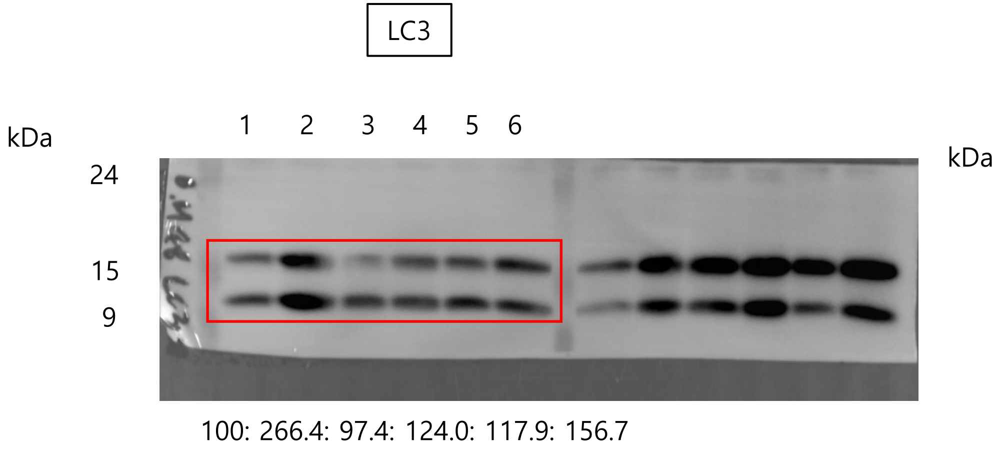

Fig 2C LC3 (8988T)

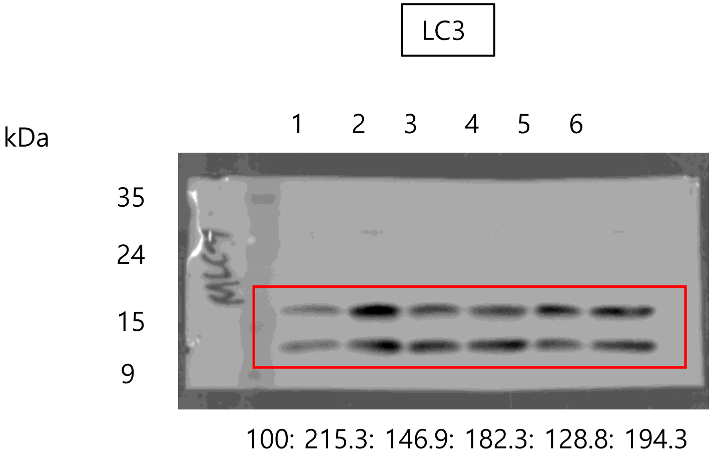

Fig 2C LC3 (MIAPACA2)

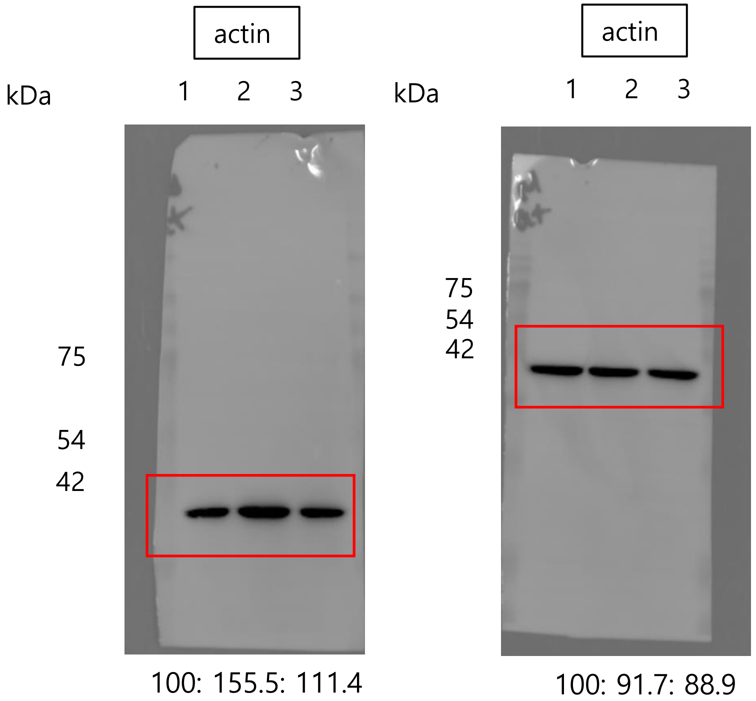

Fig 2A Actin (8988T, MIAPACA2)

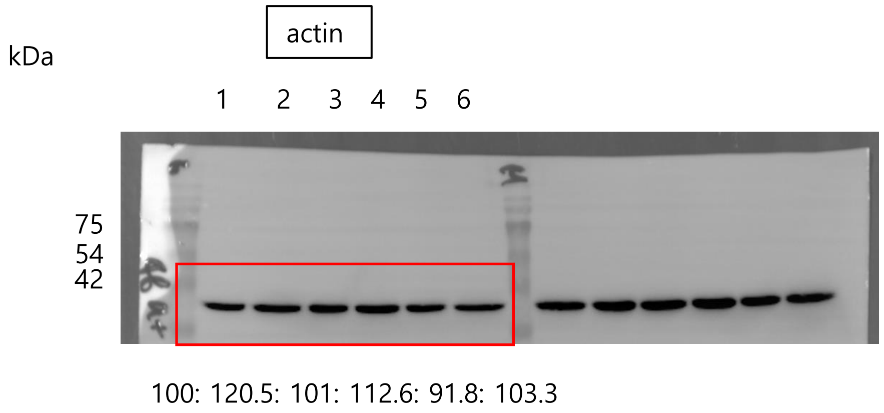

Fig 2C Actin (8988T)

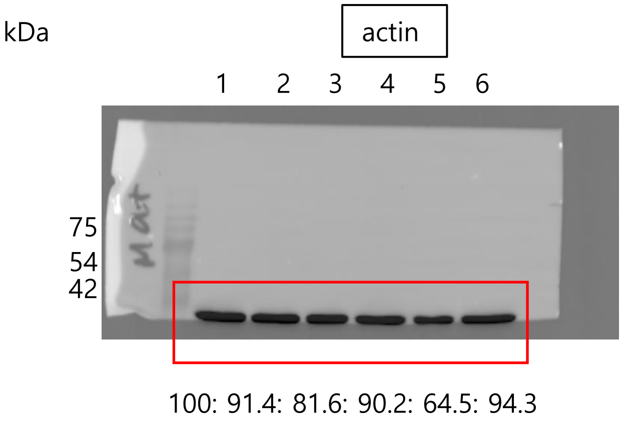

Fig 2C Actin (MIAPACA2)

Fig 5

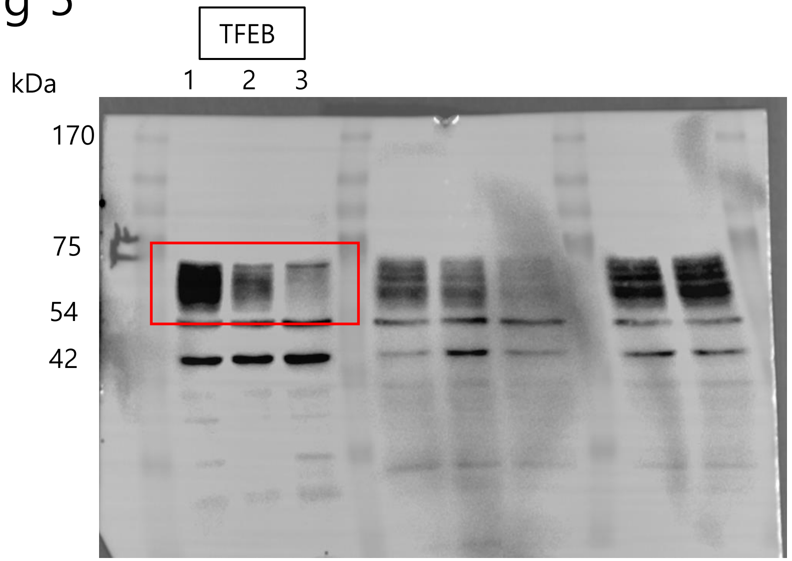

100: 52.7: 22.2

Fig 5B TFEB (8988T)

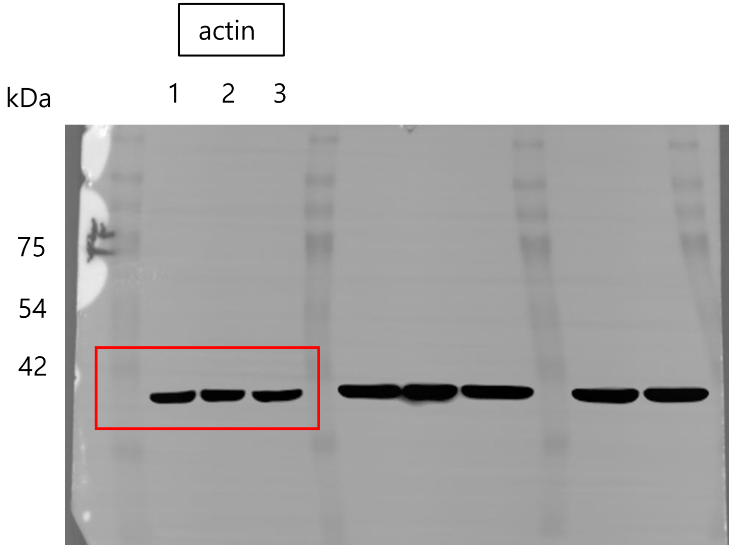

100: 96.5: 106

Fig 5B Actin (8988T)

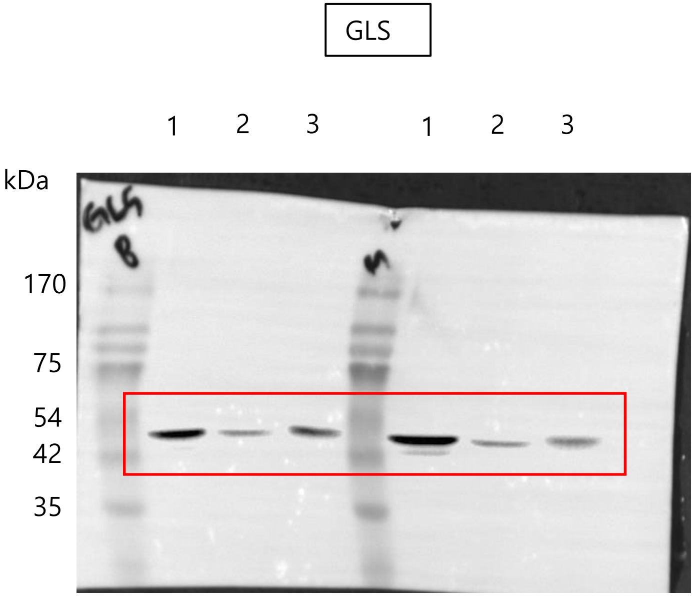

100: 44.6: 73.7 100: 33.6: 42.8

Fig 5B GLS (8988T, MIAPACA2)

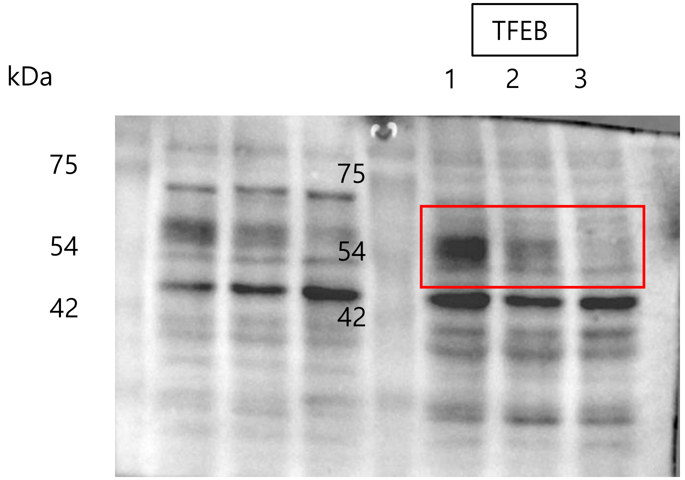

100: 64.6: 35.4

Fig 5B TFEB (MIAPACA2)

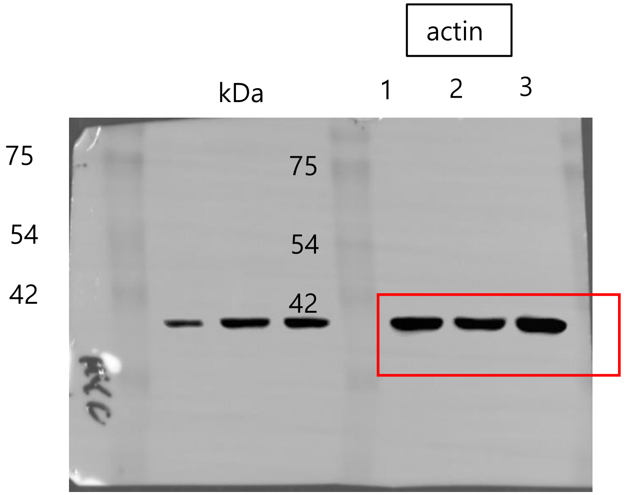

100: 87.2: 108.9

Fig 5B Actin (MIAPACA2)

Fig 5

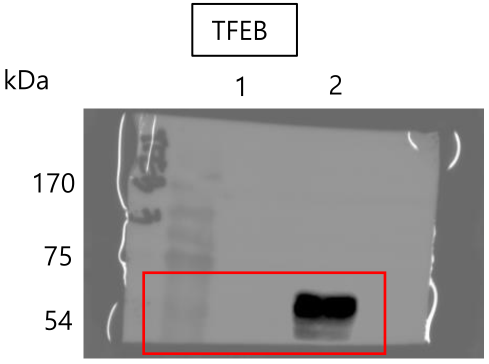

100: 9882

Fig 5D TFEB (8988T)

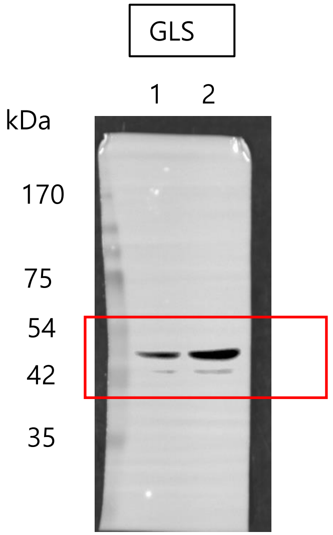

100: 158.1

Fig 5D GLS (8988T)

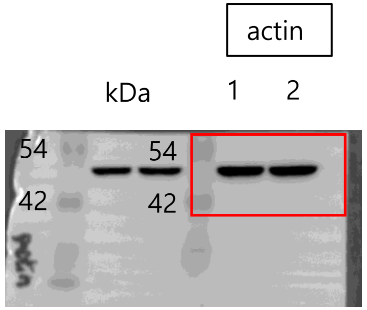

100: 104.5

Fig 5D Actin (8988T)

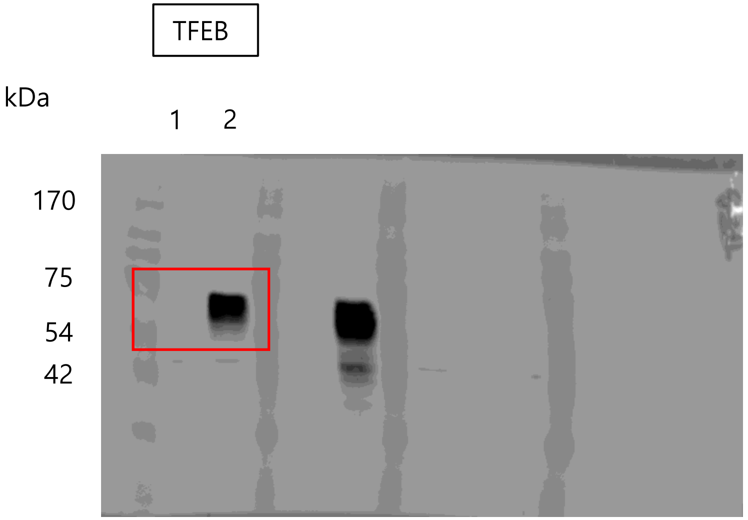

100: 1018

Fig 5D TFEB (MIAPACA2)

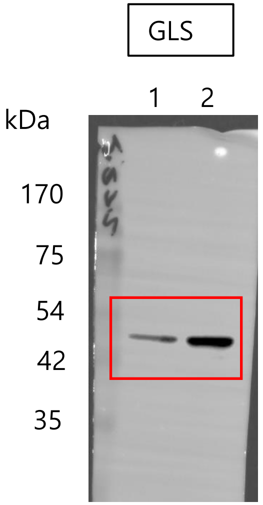

100: 167.5

Fig 5D GLS (MIAPACA2)

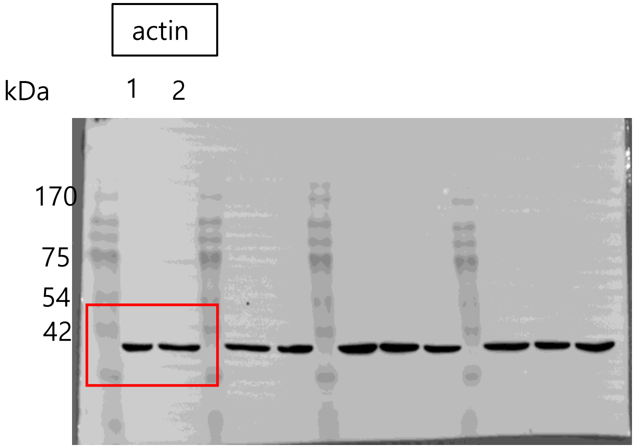

100: 99

Fig 5D Actin (MIAPACA2)

Fig 5

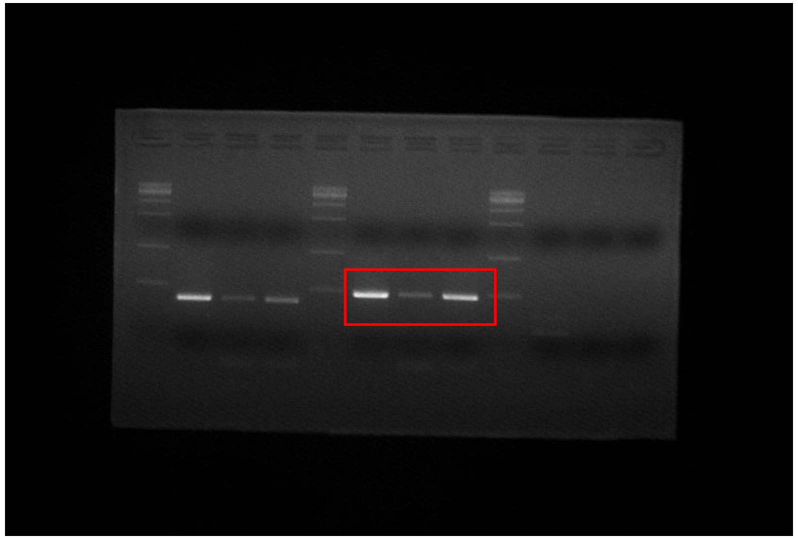

Fig 5G GLS

Fig 6

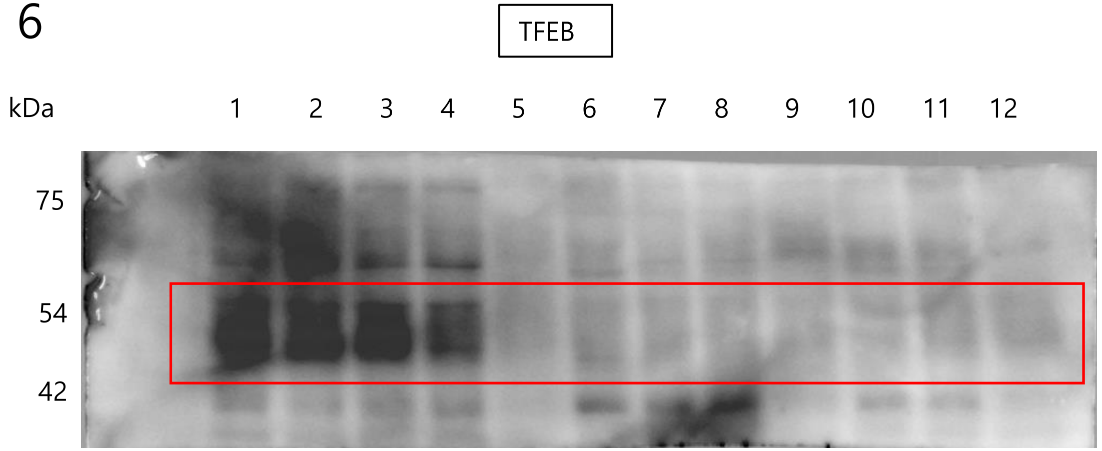

100: 89.3: 109.4: 112: 21.2: 43.3: 43.2: 58.9: 48.8: 35.5: 46.9: 53.3

Fig 6B TFEB

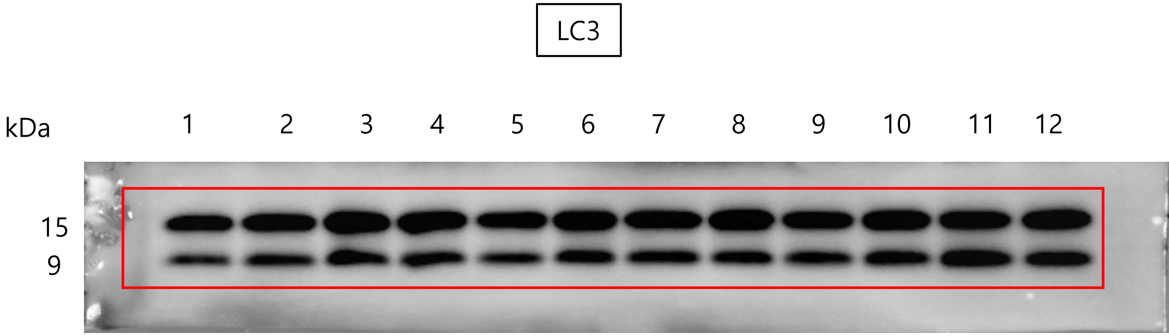

100: 125.5: 134.8: 130.1: 114.8: 121.8: 131.0: 118.5: 120.6: 135.9: 146.6: 135

Fig 6B LC3

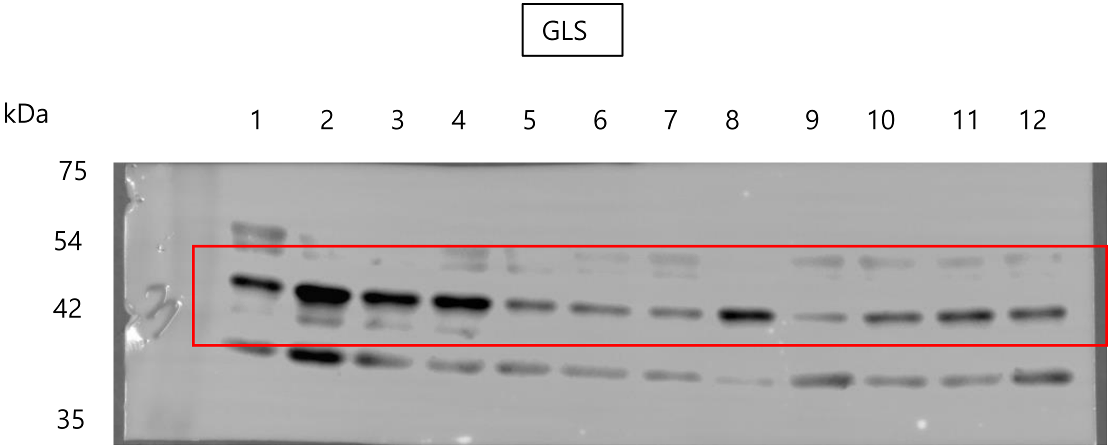

100: 207.6: 114.8: 27.5: 63: 57.8: 60.4: 90.4: 49: 68.4: 80.1: 74.8

Fig 6B GLS

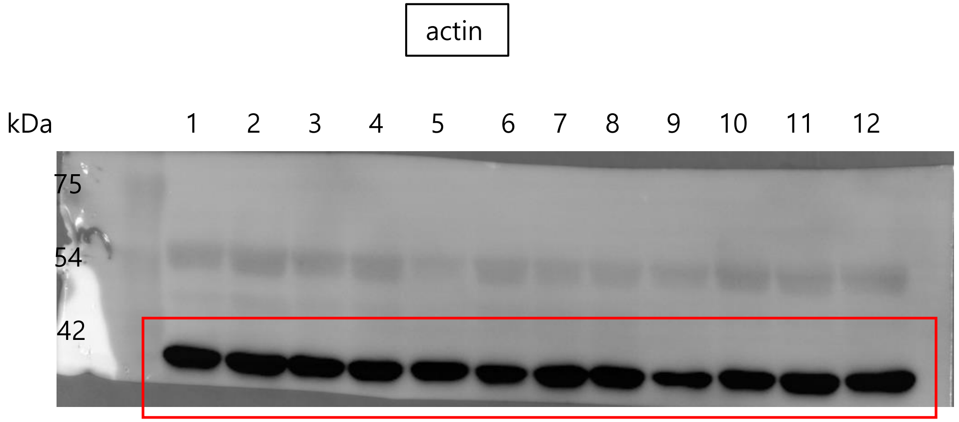

100: 106.9: 83.9: 81.4: 81.6: 63.3: 71.6: 67.2: 57.8: 65.2: 72.7: 86.3

Fig 6B Actin
